# The Influence of Gender and Year of Study on Stress Levels and Coping Strategies among Polish Dental

**DOI:** 10.3390/medicina56100531

**Published:** 2020-10-12

**Authors:** Katarzyna Mocny-Pachońska, Agata Trzcionka, Rafał J. Doniec, Szymon Sieciński, Marta Tanasiewicz

**Affiliations:** 1 Department of Conservative Dentistry with Endodontics, Faculty of Medical Science, Medical University of Silesia, Pl. Akademicki 17, 41-902 Bytom, Poland; atrzcionka@gmail.com (A.T.); martatanasiewicz@sum.edu.pl (M.T.); 2 Department of Biosensors and Biomedical Signal Processing, Faculty of Biomedical Engineering, Silesian University of Technology, Roosevelta 40, 41-800 Zabrze, Poland; rafal.doniec@polsl.pl (R.J.D.); szymon.siecinski@polsl.pl (S.S.)

**Keywords:** dental students, stress, gender differences, stress coping strategies

## Abstract

*Background and objectives*: Stress is a common term used to describe various adverse psychological conditions. Students in the dentistry field face many negative psychological outcomes. The core factors for stress among dental students are related to their training course and social contacts with peers. This research aimed to assess the stress of dental students depending on their gender and study year. *Materials and methods*: We used the Perceived Stress Scale (PSS-10) and Mini-COPE questionnaire. The surveys were conducted among 446 dental students (320 women and 126 men) at the Faculty of Medical Sciences of the Medical University of Silesia in Katowice. *Results*: For the second-year and fifth-year students, the differences in scores were statistically significant, while in both cases, men had significantly lower values on the analysed scale. The results of the Kruskal-Wallis test indicated significantly lower values on the PSS-10 scale for the third-year and fourth-year students than in first-year students. The performed statistical analysis of the data obtained from the Mini-COPE questionnaire showed significant differences between men and women in individual years of study. In the first year, women chose more often the strategies related to turning to religion (*p* = 0.007), seeking emotional support (*p* = 0.046), seeking instrumental support (*p* = 0.045) and dealing with something else *(p* = 0.029) in coping with stress than men. *Conclusions*: The highest level of stress was found among first-year dental students. Moreover, women were characterised with higher stress levels than men. Men more often use psychoactive substances and resort to a sense of humour to cope with stress. On the other hand, women turn to religion, seek instrumental and emotional support.

## 1. Introduction

Stress is a widely used term to describe various adverse psychological conditions and experiences and has numerous definitions. The definition by Lazarus and Folkman distinguishes the predecessors of stress from its consequences. This definition states that the psychological stress is a special relationship between a person and the environment, which is assessed as complicating or exceeding the abilities of an individual’s functioning and threatening to their well-being [1].

Studying the field of dentistry at a university-level possesses many negative experiences. Students experience high levels of stress, which may lead to alcohol addiction or drug use in order to alleviate it. Students often face difficulties in socialising, or they may even become depressed or irritable over time. Stress can also lead to impairments in professional skills, in the forms of reduced attention, concentration, impairments in the efficiency of their decision-making and reductions in the ability of students to build proper relationships between themselves and their patients [2,3]. The main factors responsible for stress among dentistry students are matters related to their university training courses and social contacts with their peers [2]. Stress is sometimes a desirable condition, useful in preventing boredom and insufficient stimulation. Conversely, it may cause mental or physical health disorders, stimulate the use of drugs and significantly reduce both professional and scientific effectiveness [3]. 

Medical students are forced to face situations that threaten their quality of life due to extended class hours, demanding work environments and intensive theoretical education. In contrast, dental students are required to master practical skills and obtain extensive theoretical knowledge. At the beginning of the curriculum, they undergo preclinical classes related to practical training, which require the dedication of an appropriate amount of time and depends on certain manual skills. Then, they attend meetings with patients. This situation forces a sense of responsibility within the students for the patient and their treatment as well as for any emerging complications. Another cause of stress is the exams during and after the graduation period. All these factors cause a significant level of stress among dental students, putting them at an increased risk for depression, anxiety and burnout [4,5,6]. Many authors confirm that dental students show a higher level of stress when compared to medical students and a similar level to those of other health science qualifications [7,8]. 

Numerous studies have found that gender has significant impacts on their cognitive functions, such as on their emotions, memories, perceptions, etc. [9]. Women and men show differences in memory coding, emotional experiences, facial recognition, problem-solving and decision making. Because the brain controls both cognition and behaviour, the differences between the sexes are, therefore, related to gender-dependent cerebral structures [10]. Women and men are each exposed to different types of stress and how they deal with it is, in turn, dependent upon certain cultural norms [11]. Studies have confirmed that men are more likely to respond with aggression and violence to severe stress. However, among women, stress is associated with body image issues and, as such, they tend to suffer more with eating disorders [11,12]

The aim of the study was to assess the influence of gender and year of study on psychological stress and coping strategies among Polish dental students.

## 2. Materials and Methods

### 2.1. Participants

The survey was conducted with 446 dental students from the Faculty of Medical Sciences of the Medical University of Silesia in Katowice. The study involved 91 1st-year students (67 women, 24 men) with a median age of 20 years, 98 2nd-year students (74 women and 24 men) with a median age of 21 years, 71 3rd-year students (53 women and 18 men) with a median age of 23 years, 93 4th-year students (58 women and 35 men) with a median age of 23 years, and 93 5th-year students (68 women and 25 men) with a median age of 24 years.

The data were collected anonymously, and the procedure was approved by the Ethical Commission of the Medical University of Silesia on 16 October 2018 (reference number KNW/0022/KB1/79/18). All participants gave written informed consent.

### 2.2. Methods

The surveys we used were the Perceived Stress Scale (PSS-10) and Mini-COPE questionnaire. The PSS-10 was used to assess the intensity of stress related to one’s own life experiences over the past month and was a modified version of the Perceived Stress Scale as developed by Cohen [13]. The authors conducted a survey between 20 February 2019 and 20 March 2019 after the university examination period. The PSS-10 scale contained 10 questions about various feelings related to personal problems and events, behaviours, as well as coping strategies. The respondents provided their answers by entering the correct number from a range of choices, as follows: 0–never, 1–almost never, 2–sometimes, 3–quite often, and 4–very often. Before calculating the general measure of the intensity of perceived stress, a change in the scores to the positive questions’ responses should be made, as follows: 4, 5, 7 and 8, according to the rule: 0 = 4, 1 = 3, 3 = 1 and 4 = 0. The overall score of the scale was the sum of all points, with a possible range from 0 to 40. The higher the score, the greater the severity of perceived stress. The PSS-10 scale was used to examine adults, as well as both healthy and sick people. It was mainly utilised as a self-assessment measure and can be used as a screening tool to identify people eligible for either psychological or medical assistance [14]. 

To measure the coping strategy, we used Mini-COPE, the shortened Polish version of COPE Inventory questionnaire by Carver et al. [15]. The Polish version of Mini-COPE questionnaire consisted of 28 statements, which described 14 strategies of stress management (2 statements per each strategy).

The strategies were grouped into 4 categories:

I Active coping (active coping, planning, positive reappraisal);

II Helplessness (Use of psychoactive substances; suppression of activities; self-blame);

III Seeking support (seeking emotional support; seeking instrumental support);

IV Avoidance behaviours (dealing with something else; denial; venting of emotions).

Turning to religion, acceptance and sense of humour are independent factors. The respondent marked one of 4 possible answers:

0: I almost never do this,

1: I rarely do this,

2: I often do this,

3: I almost always do it.

The survey was completed during seminar classes in accordance with the schedule for each student group. All participants completed the surveys under the same conditions: In a room with no more than 10 people in total that was insulated from noise, properly lit and ventilated, as well as containing sufficient space needed to complete the survey with comfortable seating arrangements.

### 2.3. Statistical Procedures

Statistical analyses were performed using Statistica Version 9.0 (StatSoft, Tulsa, OK, USA). For the assessment of the statistical significance of differences between consecutive years of study in terms of the respondents’ gender, we used a non-parametric independence c^2^ test. The Mann-Whitney test was used to assess the differences in values from the PSS-10 scales and Mini-COPE questionnaire in terms of the respondents’ gender for each study year. The analysis of the correlation between the PSS-10 scale and Mini-COPE strategies was performed by calculating the value of the Pearson correlation coefficient, which was then verified by a test based on the t-statistic and Student’s t-distribution. The presence of any significant differences in the PSS-10 scale responses in relation to each study year was assessed using the Kruskal-Wallis test. All statistical analyses were supplemented with the results of two post-hoc tests: The Dunn-Bonferroni and Conover tests. The significance level *p* was set to 0.05.

## 3. Results

The non-parametric c^2^ independence test did not show any statistically significant differences between the different study year groups in terms of the respondents’ gender (c^2^ = 5.262, *p* = 0.261). To confirm the reliability of the PSS-10 survey, the Cronbach’s α coefficient calculated for the selected group of respondents was 0.86 (for women α = 0.85; for men α = 0.88). Items 7, 4, 6 and 10 were suspected of being problematic for the participants. After elimination of these questions, the Cronbach’s α increased, however, this increase was not statistically significant. The answer to question 7 (‘How often, during the last month, were you able to control your exasperation?’) caused slight difficulties for women in their first, second, and fourth year of study, as well as for men in their first year. Third-year and fourth-year students experienced a problem with answering question 4 (‘How often, during the last month, were you convinced that you were able to deal with personal problems?’). Additionally, questions 6 (‘How often, in the last month, did you find you could not cope with all your responsibilities?’) and 10 (‘How often did you feel, in the last month, that you could not overcome the increasing difficulties?’) were reportedly difficult for men in their third and fifth years of study (see Table 1).

The median score of the PSS-10 scale for the whole group of respondents was 21. The average minimum value of the PSS-10 scale for all respondents was 3, and the maximum was 37. Q1 and Q3 for the participant group came to 16.5 and 26, respectively. Score distributions of the utilised PSS-10 scale, in terms of their median, minimum and maximum, as well as the Q1 and Q3 for each year of study, are presented in Table 2.

**Figure 1:**
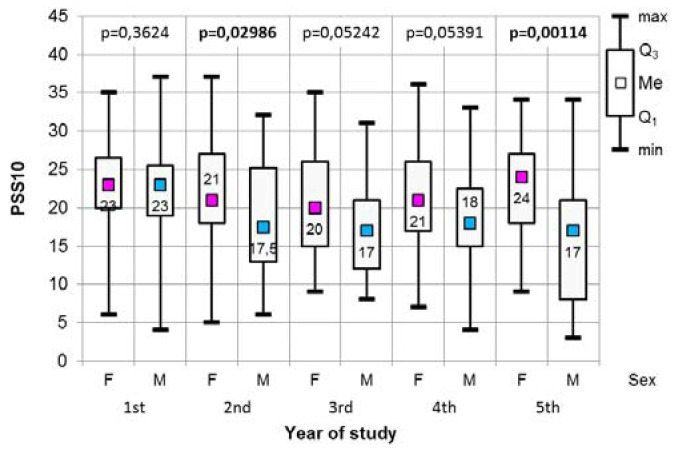
The results of the Mann-Whitney test used to assess the significant differences in the PSS-10 values in relation to respondents’ gender, including their year of study.

The results of the Mann-Whitney test, which was used to assess any significant differences in the values of the answers to the PSS-10 scale, in terms of the respondents’ gender in each study year, are presented in Figure 1. The difference between the second and fifth-year students was statistically significant, while in both cases men obtained significantly lower values in the analysed scale. In addition, in both the third and fourth years, there was a statistically significant difference found between women and men. Women, in these specific study years, were characterised by a higher level of perceived stress.

**Figure 2:**
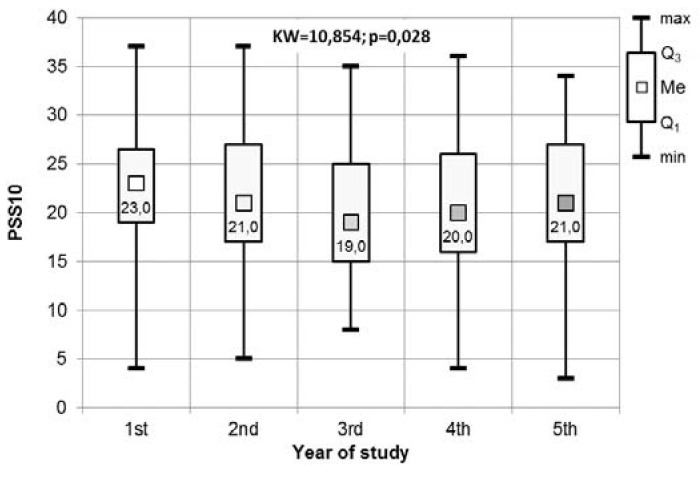
The results of the Kruskal-Wallis test used to assess the significant differences in the PSS-10 scale in relation to participants’ year of study.

Figure 2 presents the results of the Kruskal-Wallis test used to assess the presence of any significant differences in the PSS-10 scale in relation to participants’ year of study. These differences were shown to be statistically significant and, as such, the results of the statistical analysis were supported by those of the two post-hoc tests: The Dunn-Bonferroni and Conover tests. The first of these found that the results of the Kruskal-Wallis test were due to the significantly lower values on the PSS-10 scale for those in their third year compared to participants in their first year. According to the Conover test, there was also a significant difference in the comparison of the fourth and first years (see Figure 2 and Table 3).

We observed significant differences between the results from the Mini-COPE questionnaire between male and female subjects in each year of study (Table 4). In the first year, women chose the strategies of coping with stress related to turning to religion (*p* = 0.007), seeking emotional support (*p* = 0.046), seeking instrumental support (*p* = 0.045) and dealing with something else (*p* = 0.029) significantly more often than men. Results shown in Table 4 suggest that men who studied in the second year chose to use psychoactive substances (*p* = 0.004) and escape into their sense of humour (*p* = 0.033) to cope with stressful situations. On the other hand, women more often turned to seeking instrumental support (*p* = 0.018), venting of emotions (*p* = 0.005) and blaming themselves (*p* = 0.046).

The third-year male students coped with stressful situations by escaping into humour (*p* = 0.043). Denial (*p* = 0.029) and self-blame (*p* = 0.001) strategies were chosen more often by third-year female students than men. The most prominent differences observed in female fourth-year students were in coping with stress by turning to religion (*p* = 0.04), seeking emotional support (*p* = 0.01), seeking instrumental support (*p* = 0.008) and venting of emotions (*p* = 0.04). There were no significant differences in choosing strategies for coping with stress between female and male fifth-year students (see Table 4).

The analysis of the correlation between the values of the PSS-10 scale and subsequent strategies of the Mini-COPE questionnaire in relation to the subsequent years of study is presented in Table 5. Within the “planning” strategy, positive correlations with the results of the PSS-10 were obtained at first-year students (*p* = 0.001), and fourth-year students (*p* < 0.001).

In the case of positive reappraisal, positive correlations were obtained for third-year students (*p* = 0.018) and fifth-year students (*p* = 0.001). The “Acceptance” strategy had a significant positive correlation with the PSS-10 scale in all years of study, reaching successively from the first year to the fifth year. The p-values were: 0.003, 0.011, 0.004, 0.001 and 0.034.

Only in the first year of studies, the sense of humour strategy did not correlate with the PSS-10 scale. The obtained p values for the 1–3 year of study: 0.030 (1st) 0.006 (2nd) 0.037 (3rd) for the seeking instrumental support strategy strongly correlated with the PSS-10. Strategy: Dealing with something else, positively correlated only in the fifth year (*p* = 0.007). A statistically significant correlation between the scales was also obtained for the venting of emotions strategy for the first-year students (*p* = 0.040), fourth-year students (*p* = 0.010) and fifth-year students (*p* = 0.044).

Positive correlation for the strategy “use of psychoactive substances” was not observed only in the group of fourth-year students. The suppression of activities strategy had a significant positive correlation for the first-year (*p* = 0.020) and fifth-year students (*p* < 0.001). For the “self blame” strategy, a positive correlation with the PSS-10 scale was observed on first (*p* = 0.009), fourth (*p* = 0.007) and fifth-year students (*p* = 0.002). A significant negative correlation was observed only in one case: For the “planning” strategy in third-year students (r = −0.615, *p* ≤ 0.001).

## 4. Discussion

A review of the literature indicates that university students, in particular, have an increased susceptibility to experiencing stress. Studies have shown that the time needed to complete their qualifications is particularly important in the lives of young adults. This time is sometimes referred to as a formative period and, thus, may have long-term repercussions on peoples’ later health and mental statuses [16].

Undergraduate dental education in Poland is a five-year program. The first year primarily consists of theoretical classes, wherein students acquire general medical and dental knowledge. Students generally start their education immediately after graduating from high school [17]. The level of competition in this program is often very high and is compounded by the large amount of new information that needs to be learned. These facts helped to inform the current research. Based on the survey results, the highest level of experienced stress was experienced by first-year students.

In contrast to the findings of our research, Saddki et al. reported higher values of perceived stress in senior students (i.e., those in their fourth and fifth years) who undergo clinical classes [18] on 234 undergraduate dental students, using the PSS-10 questionnaire. Jowkar et al. examined a group of 150 dental students, in which the lowest and highest stress values were obtained from those in their fifth and sixth years of study, respectively. This study also utilised the Depression, Anxiety and Stress Scale (DASS-21) survey, which showed no differences between the study years examined [19]. In Silverstein et al. [20], a study on the stress levels of 296 first-year dental students, the students completed the Dental Environment Stress (DES) and PSS questionnaires twice: At the beginning of the academic year and at the end. In total, 205 respondents completed the surveys.

Silverstein et al. found that stress among students increased after a year of studying [20]. At the beginning, there were clear differences between women, those not in a relationship, and younger people, in that they experienced significantly more stress. However, one year after the first study year, these differences were not observed except for an overall increase in their perceived stress. The authors suggested that first-year students should be familiar with stress-reducing techniques. This phenomenon is probably associated with the fact that first-year students experience unique changes in their lives, including moving into a new environment, leaving friends and family, the need to make new acquaintances, assimilation of new knowledge, and taking responsibility for their own finances. All these factors would affect the increased levels of stress that they experience.

The findings in [20] were confirmed in our study. Female students coped with stress more often by turning to religion, seeking emotional and instrumental support and dealing with something else. This indicates that women react more emotionally when faced with a stressful situation. There were no significant differences between men and women in choosing the active form of coping with stress (active coping, planning, positive reappraisal).

Ersan et al. [21] in a study on 100 preclinical dental students using dental environment stress (DES), perceived stress (PSS), general self-efficacy (G-SES) and brief coping scales (Brief-COPE), showed that preclinical students have moderate levels of stress in contrast with students having clinical work with patients whose stress level is high. Social stressors affected more significantly male students than female students. The most common stress coping strategies were planning and using drugs. As in our study, women chose seeking for instrumental support significantly more often than men. Demographic factors influenced both the level of perceived stress and coping strategies.

Sanchez-Conde et al. conducted a study that aimed to analyse the psychological profile of nursing students and their relationship with the psychophysiological reaction of anticipatory anxiety in the early stages of clinical practice and to analyse the habituation response at the psychophysiological level. One of the tools used in the study was the PSS-10 scale. The obtained results (27.4 ± 8.4) were similar to ours (median 21). The first hypothesis of the study was confirmed—the participants had a large anticipatory anxiety response. On the other hand, the second hypothesis concerning the habituation process was not confirmed. Higher social perception isolation was significantly associated with higher objective (sympathetic modulation) and subjective (perceived stress) stress responses [22].

Another study was conducted by Cavallo et al. [23,24] with a group of 278 students studying at the Department of Science at the University of Salerno, Italy. Here, the authors assessed the correlations between awake and asleep bruxism. In the study, the PSS scale was used to assess stress, with the occurrence of bruxism being measured using the Fonseca questionnaire. Students exhibited a higher level of both bruxism and stress when compared to the general population. The mean score of the PSS scale in this study was 32.2, with the scores for the general population typically range from 12 to 14 [23]. In our study, the median score for the whole group of respondents came to 21, with women, specifically, being characterised by higher levels of stress, which was especially noticeable for those living with their families. The authors emphasise that higher values for perceived stress may result from higher expectations of one’s family. In addition, we observed that women who did not smoke or use drugs experienced higher levels of stress. Nevertheless, a positive correlation between awake bruxism and stress was found only in men in the aforementioned study [24].

Z. H. Al-Sowygh [25] conducted a survey of 556 dental students from the first to the fifth years of study. The highest level of stress was felt by students of the fourth year, followed then by those in their first. The second-year students obtained the lowest values along the PSS scale. We confirmed that women experienced significantly higher levels of stress than men. The most significant differences between men and women were observed in choosing to cope with stress by denial, self- blame and behavioural disengagement. In Al-Sowygh study, men chose self-blame strategies significantly more often than women. The most common ways of coping with stress in the analysed group of students were active coping, planning, religion and acceptance [25]. Religion was also one of the most frequently chosen ways of coping with stress by first and fourth-year female students

The PSS-10 scale was also used in the research conducted by Zinurova et al. in their assessment of perceived stress in 505 first-year postgraduate pharmacy residents across the United States. Women were found to have higher levels of stress than men, similar to the results of our study. There were no significant differences between single and married residents in stress levels. Conversely, residents with children had higher stress values than those having no children. Other factors affecting peoples’ perceived stress levels included time pressures, work overload, and fear of making errors [26].

An important aspect of research determining the level of stress with the use of various psychological tools (scales, questionnaires) is an attempt to link the results obtained with the physiological response of the organism. A study by Ramirez-Adrados et al. showed that the final exam in physiotherapy students induced a large anticipatory anxiety response. However, the parameters of autonomous modulation are not related to the completion of the diploma thesis. Interestingly, before and during the thesis defence, the HRV (heart rate variability) parameters in 110 volunteers remained at a low level [27].

Numerous other authors also confirmed that women experienced higher levels of stress and anxiety [28,29,30,31]. This is due to significant psychological differences between the genders, in that women are more likely to show their worries and emotions [32]. In the current research, women, especially those in their second and fifth years, experienced higher stress values than did their peers. Third- and fourth-year female students also demonstrated higher stress values when compared to men, but these were only on the border of statistical significance. Similar results were also obtained by Saddki et al., as well as by other authors [18,33,34,35]. 

Higher levels of perceived stress among women were probably due to the fact that they, when compared to men, were more susceptible to both physical and emotional problems such as depression and fatigue [26]. In addition, marital status seems to be important in the development of perceived stress. Single students were reportedly more stressed in the context of factors affecting their future careers and the responsibility needed for their studies. They were less confident in making decisions. Similarly, single females and men who were separated/widowed/divorced showed higher levels of stress when compared to their married peers. 

Married students only experienced greater stress than single ones due to their university training because marriage provides massive social support and positively influences one’s academic career, as well as acting as a buffer during times of stress [36]. Difference between men and women were also found in their ability to feel empathy. The study conducted by Mocny-Pachońska et al. [37] with the group of 100 polish dental students revealed significant difference between men and women of fifth year.

To summarise, the most significant problem in measuring the level of stress is ensuring objectivity. In a pilot study, Mocny-Pachońska et al. [38] proposed using physiological signals acquired by wearable devices, such as smart glasses. However, this approach requires further research.

### Limitations of this Study

This study has some limitations that need to be considered when interpreting its findings. 

The survey research was conducted among dental students of only one Medical University. An interesting point of focus would be the comparison of stress levels between dental students and those in other university fields; for example, those in either the university of technology or philology. 

## 5. Conclusions

The highest level of stress was represented by dental students currently in their first year of study. Women were characterised with higher stress levels than men. In our study, the PSS-10 scale turned out to be a useful stress screening tool in measuring anxiety among dental students. To cope with stress, men use psychoactive substances and resort to a sense of humour more often than women. Women, on the other hand, turn to religion, seek instrumental and emotional support and deny and blame themselves more often than men.

### Practical Application

The results of conducted research should be important for policymakers and educators alike to understand the stress-related associations outlined by this study in their daily professional practice to ensure their students are receiving optimal support and guidance.

## Figures and Tables

**Table 1:** Relialibity of the PSS-10 scale.

PSS-10		1–10			Item Elimination	
Year	Group	α	α_st_	R_av_	Item	α
I	Total	0.851	0.850	0.361	7	0.855
	Female	0.826	0.825	0.320	7	0.831
	Male	0.898	0.898	0.468	7	0.900
II	Total	0.878	0.879	0.420	7	0.881
	Female	0.881	0.880	0.424	7	0.887
	Male	0.860	0.863	0.386	−	−
III	Total	0.834	0.831	0.330	4	0.835
	Female	0.822	0.819	0.312	4	0.827
	Male	0.863	0.862	0.384	6	0.890
IV	Total	0.862	0.864	0.388	−	−
	Female	0.872	0.873	0.407	7	0.873
	Male	0.838	0.841	0.346	4	0.840
V	Total	0.884	0.885	0.434	7	0.884
	Female	0.843	0.841	0.346	−	−
	Male	0.906	0.911	0.505	10	0.913
I-V	Total	0.865	0.865	0.391	−	−
	Female	0.851	0.851	0.363	7	0.855
	Male	0.879	0.880	0.424	−	−

PSS-Perceived Stress Scale; α_st-_ standardised alpha; R_av-_ average correlation coefficient.

**Table 2:** Values of the minimum, maximum, median and Q1 and Q3 of scores in each year of study.

	Year of Study					
PSS-10	1st	2nd	3rd	4th	5th	Total
Min	4	5	8	4	3	3
Q_1_	19	17	15	16	17	16.25
Me	23	21	19	20	21	21
Q_3_	26.5	27	25	26	27	26
Max	37	37	35	36	34	37

**Table 3:** The results of the two post-hoc tests: The Dunn-Bonferroni and Conover tests.

		Dunn-Bonferroni				
Conover	Year	I	II	III	IV	V
	I		0.47	0.008	0.12	0.83
	II	0.08		0.50	1.00	1.00
	III	0.01	0.08		1.00	0.30
	IV	0.04	0.29	0.16		1.00
	V	0.11	0.39	0.06	0.21	

**Table 4:** Strategies of coping with stress in the examined group of students obtained from the MINI-COPE questionnaire in relation to gender (Mann-Whitney test).

Strategies		Female	Male	*p*
First year of study				
6: Turning to Religion	Median	0.5	0.0	0.007
	Quartiles	0.0–1.5	0.0–0.6	
	Min-Max	0.0–3.0	0.0–2.0	
7: Seeking emotional support	Median	2.5	2.0	0.046
	Quartiles	1.5–2.5	1.5–2.5	
	Min-Max	0.0-3.0	0.0–3.0	
8: Seeking instrumental support	Median	2.0	2.0	0.045
	Quartiles	1.5–3.0	1.0–2.0	
	Min-Max	0.0-3.0	0.5–3.0	
9: Dealing with something else	Median	2.0	1.5	0.029
	Quartiles	1.0–2.0	1.0–1.6	
	Min-Max	0.0–3.0	0.0-2.5	
Second year of study				
5: Sense of humor	Median	1.0	1.3	0.033
	Quartiles	0.5–1.5	0.5–2.0	
	Min-Max	0.0–3.0	0.0–3.0	
8: Seeking instrumental support	Median	2.0	1.8	0.018
	Quartiles	1.5–2.5	1.0–2.5	
	Min-Max	0.5–3.0	0.5–2.5	
11: Venting of emotions	Median	1.5	1.5	0.005
	Quartiles	1.5–2.0	1.0–1.6	
	Min-Max	0.0–3.0	0.5–3.0	
12: Use of psychoactive substances	Median	0.0	1.0	0.004
	Quartiles	0.0–1.0	0.0–2.0	
	Min-Max	0.0–3.0	0.0-2.5	
14: Self blame	Median	2.0	1.25	0.046
	Quartiles	1.0–2.0	1.0–2.0	
	Min-Max	0.0–3.0	0.0–2.5	
Third year of study				
5: Sense of humor	Median	1.0	1.5	0.043
	Quartiles	0.5–1.5	0.6–2.0	
	Min-Max	0.0–3.0	0.0–2.5	
10: Denial	Median	0.5	0.25	0.029
	Quartiles	0.0–0.5	0.0–1.0	
	Min-Max	0.0–3.0	0.0–2.0	
14: Self blame	Median	1.5	1.0	0.001
	Quartiles	1.0–2.0	1.0–1.3	
	Min-Max	0.0–3.0	0.0–2.5	
Fourth year of study				
6: Turning on Religion	Median	1.0	0.0	0.04
	Quartiles	0.0–1.8	0.0–1.7	
	Min-Max	0.0–3.0	0.0–3.0	
7: Seeking emotional support	Median	2.5	2.0	0.01
	Quartiles	1.5–3.0	1.0–2.0	
	Min-Max	0.5–3.0	0.0–3.0	
8: Seeking instrumental support	Median	2.0	2.0	0.008
	Quartiles	1.5–3.0	1.5–2.0	
	Min-Max	0.0–3.0	0.0–2.5	
11: Venting of emotions	Median	2.0	1.5	0.04
	Quartiles	1.5–2.0	1.3–2.0	
	Min-Max	0.0–3.0	0.5–2.5	

**Table 5:** The results of the correlation analysis between the PSS-10 scale and Mini-COPE strategies.

Strategies		Year of Study					Total
		1st	2nd	3rd	4th	5th	
1: Active coping	r*	−0.004	0.078	0.015	−0.124	−0.010	−0.009
	*p*	0.973	0.448	0.903	0.237	0.921	0.845
2: Planning	*r*	0.355	–0.031	−0.615	0.590	0.163	0.130
	*p*	0.001	0.764	<0.001	<0.001	0.118	0.006
3: Positive reappraisal	*r*	0.026	0.203	0.281	0.137	0.327	0.199
	*p*	0.807	0.046	0.018	0.190	0.001	<0.001
4: Acceptance	*r*	0.304	0.256	0.337	0.337	0.220	0.279
	*p*	0.003	0.011	0.004	0.001	0.034	<0.001
5: Sense of humor	*r*	0.108	0.443	0.389	0.303	0.219	0.287
	*p*	0.309	<0.001	0.001	0.003	0.035	<0.001
6: Turning to Religion	*r*	0.021	0.025	0.083	−0.061	0.179	0.047
	*p*	0.844	0.810	0.492	0.562	0.086	0.317
7: Seeking emotional support	*r*	−0.048	0.090	−0.093	−0.145	−0.089	−0.053
	*p*	0.651	0.377	0.439	0.165	0.398	0.261
8: Seeking instrumental support	*r*	0.228	0.274	0.248	0.061	0.142	0.185
	*p*	0.030	0.006	0.037	0.562	0.175	<0.001
9: Dealing with something else	*r*	0.047	−0.071	0.169	0.167	0.279	0.121
	*p*	0.656	0.486	0.158	0.110	0.007	0.011
10: Denial	*r*	0.105	0.135	0.069	0.071	0.153	0.116
	*p*	0.320	0.184	0.568	0.497	0.144	0.014
11: Venting of emotions	*r*	0.216	0.183	0.140	0.267	0.209	0.205
	*p*	0.040	0.072	0.244	0.010	0.044	<0.001
12: Use of psychoactive substances	*r*	0.383	0.206	0.310	0.197	0.283	0.265
	*p*	<0.001	0.042	0.008	0.059	0.006	<0.001
13: Suppression of activities	*r*	0.244	−0.060	0.088	0.118	0.360	0.152
	*p*	0.020	0.560	0.465	0.260	<0.001	0.001
14: Self blame	*r*	0.272	−0.040	−0.165	0.277	0.321	0.160
	*p*	0.009	0.694	0.170	0.007	0.002	0.001

r*—Pearson correlation coefficient.
